# Chromosomal Abnormalities in Couples Experiencing Recurrent Implantation Failure in West of Iran: A Case–Control Study

**DOI:** 10.1002/mgg3.70137

**Published:** 2025-09-05

**Authors:** Atefeh Asgari, Amir Mohammad Salehi, Fatemeh Shahbazi, Safieh Ghahremani, Ebrahim Kamrani Saleh

**Affiliations:** ^1^ Research Center for Molecular Medicine, Institute of Cancer, Avicenna Health Research Institute (AHRI) Hamadan University of Medical Sciences Hamadan Iran; ^2^ Student Research Committee, Hamadan University of Medical Sciences School of Medicine Hamadan Iran; ^3^ Department of Epidemiology, School of Public Health Hamadan University of Medical Sciences Hamadan Iran; ^4^ Modeling of Noncommunicable Diseases Research Center, Health Sciences & Technology Research Institute Hamadan University of Medical Sciences Hamadan Iran; ^5^ Cytogenetic Laboratories, Shahid Beheshti Hospital Hamadan University of Medical Sciences Hamadan Iran

**Keywords:** assisted reproductive technique, chromosomal abnormalities, genetic, infertility, IVF, karyotyping

## Abstract

**Background:**

Recurrent Implantation Failure (RIF) is defined as the inability to establish pregnancy despite high‐quality embryo transfer after the application of at least three consecutive in vitro fertilization (IVF)/intracytoplasmic sperm injection‐embryo transfer procedures. Chromosomal abnormalities are one of the primary reasons for pregnancy failure, miscarriage, and birth defects in both natural conception and IVF pregnancies. This study was to evaluate the incidence of chromosomal abnormalities in peripheral blood samples from 100 couples who experienced RIF.

**Methods:**

Chromosomal structure analysis was conducted on peripheral blood samples from 100 couples who experienced RIF between 2018 and 2022. Additionally, cytogenetic assessments were conducted on 200 healthy individuals without clinical issues to ensure the accuracy. The GTG‐Banding technique was employed in our research.

**Results:**

Out of the 200 individuals who faced RIF, six (3%) exhibited chromosomal abnormalities, comprising five (83.3%) men and one (16.6%) woman. Translocation was the main type of autosomal structural abnormalities; also, we found one inversion and one pstk ‐ (population polymorphism). Conversely, no chromosomal abnormalities were detected in the control group. We found chromosomal abnormalities in 3% of study participants who had experienced RIF.

**Conclusion:**

Chromosomal abnormalities significantly contribute to RIF. Therefore, it is imperative to conduct cytogenetic screening for both partners before initiating any assisted reproductive technology procedures.

## Graphical abstract

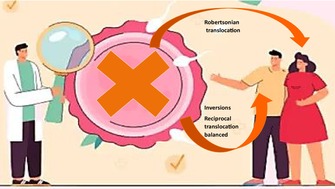

**Abbreviations**
IVF: In Vitro Fertilization
PHA: phytohemagglutinin
RIF: Recurrent implantation failure

## 1 Introduction

Infertility is defined as the inability to conceive after 1 year of regular intercourse without the use of contraception methods or the failure to produce a successful pregnancy. Infertility is one of society's major issues (Ahmadi Mazjin et al. 2015). The widespread occurrence of infertility in society emphasizes the significance of this issue. In Iran, between 10% and 15% of couples struggle with infertility (Akhondi et al. 2019).

Although in vitro fertilization (IVF) has revolutionized infertility therapy, many couples experience embryo transfer failure (Bashiri et al. 2018). The success rate of live births after multiple IVF attempts can be low, leading to financial, physical, and emotional strain for couples (Aimagambetova et al. 2020). Recurrent implantation failure (RIF) (OMIM: 614389) is a challenge for both couples and fertility specialists (Petrovic et al. 2024).

RIF is defined as the inability to establish pregnancy despite high quality embryo transfer after application of at least three consecutive IVF/intracytoplasmic sperm injection‐embryo transfer procedures. The etiology of this condition is unclear and probably results from multiple factors (Turan et al. 2015). Chromosomal abnormalities are one of the primary reasons for pregnancy failure, miscarriage, and birth defects in both natural conception and IVF pregnancies (Petrovic et al. 2024).

It is common for couples to undergo IVF without undergoing a thorough genetic analysis, which could reveal genetic causes such as chromosomal aberrations that may significantly contribute to their RIF (Mierla et al. 2015). Several studies have reported chromosomal aberrations in 2%–7% of infertile couples, and some data suggest even higher percentages, especially in cases of male infertility (Petrovic et al. 2024; Etemadi and Amiri 2013; Kocaaga et al. 2022).

To date, studies conducted in Iran on the genetic causes of RIF have been limited. Therefore, this study aimed to determine parental chromosomal abnormalities related to RIF in the west of Iran.

## 2 Materials and Methods

### 2.1 Study Design

The STROBE reporting guidelines were used in the design of this study. All couples participating in this study were thoroughly informed about its purpose, procedures, and potential risks. Written informed consent was obtained from all participants before any data was collected or RIF‐related procedures were carried out.

### 2.2 Participants

From April 2018 to March 2022, we conducted this case–control study on 200 patients (100 couples) with RIF. We thoroughly documented the medical histories of all subjects. To ensure the validity of our results, cytogenetic tests were conducted on 200 healthy individuals devoid of any clinical issues who served as controls. The case and control groups are matched based on gender and their place of residence. The case group included all couples who attended infertility clinics in Hamadan County specifically for evaluation of RIF during the study period. The control group consisted of women who were present at the same clinics during the same timeframe for non‐infertility‐related reasons (e.g., routine gynecological care). After receiving a detailed explanation of the study objectives and providing written informed consent, eligible women in the control group and their male partners were invited to voluntarily undergo complementary karyotype analysis at no cost. This approach allowed for the recruitment of control participants from a comparable clinical setting while ensuring voluntary participation and ethical compliance. Given the nature of the case–control study and the fact that the highest statistical power is obtained with a 1:1 case‐to‐control ratio, we also considered 100 couples for the control group.

The inclusion criteria for this study included women who did not have any intrauterine conditions, such as intrauterine adhesions, submucous myoma, or any other uterine abnormalities. This was determined through diagnostic hysteroscopy, serology, and hysteroscopy. Men with thrombophilia autoimmunity were also included if clinical and andrological examinations revealed no issues. Couples aged between 25 and 35 years, who had at least two unsuccessful IVF attempts, were also included in this study.

The study had certain exclusion criteria in place. Individuals who faced difficulties in diagnosing endometriosis, submucous myoma, other uterine abnormalities, thrombophilia, autoimmunity, and those who were outside the age range of 25–35 and had used other assisted reproductive methods were not included. Additionally, women who experienced total fertilization failure and partial fertilization failure in the transition cycle were also excluded from the study.

### 2.3 Chromosomal Studies

G‐banding was utilized for karyotyping through the peripheral lymphocyte culture approach. For 72 h, the peripheral blood lymphocytes were cultured in RPMI 1640 medium (Gibco‐BRL and phytohemagglutinin (PHA)) with l‐glutamine and 20% fetal bovine serum. After 72 h, the cultivated cells were taken, and the chromosomes were linked using the trypsin‐giemsa procedure. At least 20 metaphases were examined for each patient, and 30 to 40 cells were investigated in each case. All chromosomal anomalies were reported in compliance with international guidelines (Meng et al. 2023; Liehr 2021). When photographing images, the desired cells were captured when fractures or other chromosomal anomalies emerged under the microscope. In describing abnormalities, chromosome polymorphisms or variations, as well as fragile spots, were disregarded. This study does not focus on specific genes; therefore, OMIM accession numbers are not provided. OMIM (Online Mendelian Inheritance in Man) is a reliable and comprehensive online database in the field of human genetics that collects information about genes and hereditary diseases. Each gene or hereditary disease in this database has a unique number, called an OMIM accession number. This number helps researchers to accurately and uniquely reference information about a specific gene or disease and avoid ambiguity in naming.

### 2.4 Statistical Analysis

Descriptive categorical and continuous predictors were expressed as number (%) and mean (standard deviation) according to the study groups, respectively, and were tested using chi‐square as appropriate.

### 2.5 Ethical Considerations

All adult subjects provided informed consent, and this study received approval from the Hamadan University of Medical Sciences Ethics Committee (IR.UMSHA.REC.1400.495).

## 3 Results

The mean age of these individuals was 32.0 ± 1 while the mean infertility period was 4.0 ± 1 years. The frequency of karyotype abnormalities in this study was 3% as summarized in Table 1.

**TABLE 1:** Somatic karyotypes in couples referred for infertility treatment by IVF.

Number	Gender	Patient age (years)	History	Karyotype	Diagnosis
1	Male	34	4 IVF	46,XY,t(2;7)(p21;q15)	Reciprocal translocation balanced
2	Male	29	2 IVF	46XY,inv.(1)(p13; p12)	Inversions
3	Male	32	3 IVF	46,XY,t(7;14)(q22;q22)	Reciprocal translocation balanced
4	Female	33	4 IVF	46,XX,13pstk—	Satellites
5	Male	30	3 IVF	46,XY,t(13;18)(q12;p11.3)	Reciprocal translocation balanced
6	Male	29	2 IVF	46,XY,t(13;18) (q13;p11.3)	Reciprocal translocation balanced
7	Female	28	2 IVF	46,XX,t(15;22)(q10; q10)	Robertsonian translocation

**FIGURE 1:**
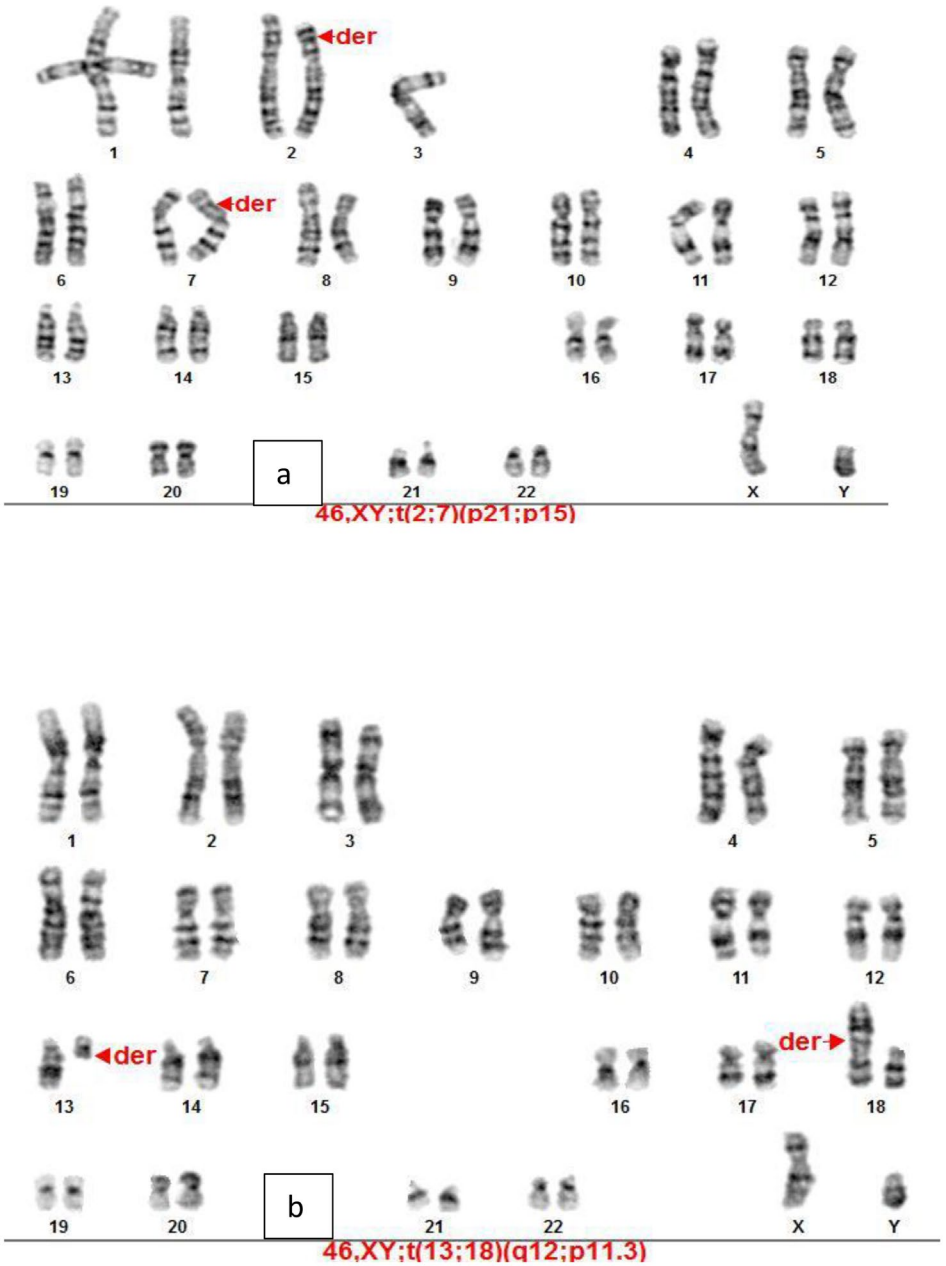
GTG banded karyotype 1. Arrows show a t (2; 7) (p21; q15) translocation (G‐banded karyotype) in an infertile man. (a) GTG banded karyotype 4. Showing a t (13; 18) (p12; q11.3) translocation (G‐banded karyotype) in an infertile man (b).

**FIGURE 2:**
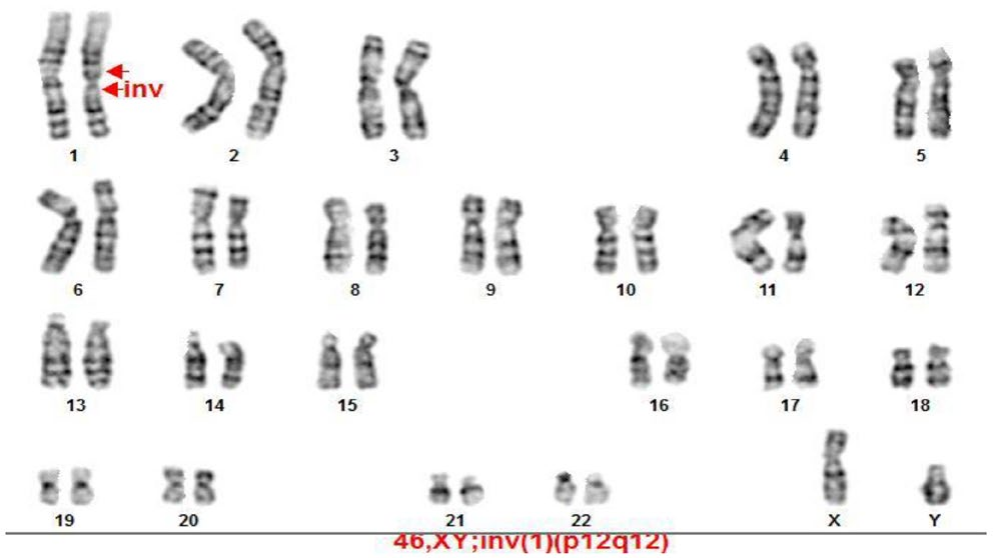
GTG banded karyotype2. Showing an inv. (1) (p12; q12) (G‐banded karyotype) in an infertile man.

**FIGURE 3:**
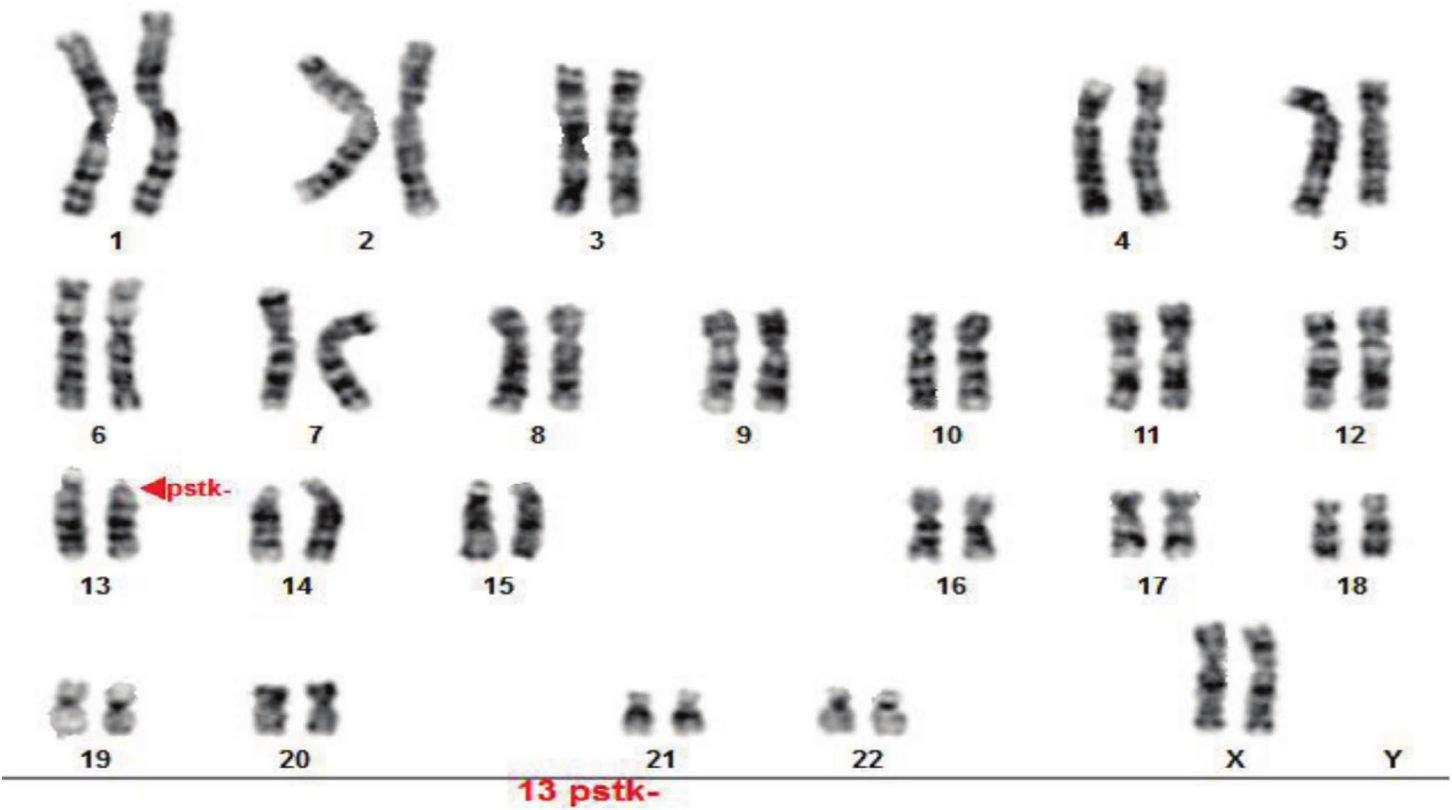
GTG banded karyotype 3. Showing a 13 pstk– (G‐banded karyotype) in an infertile female.

The results of the experiments were such that we observed six cases of chromosomal abnormalities among the 200 karyotypes examined in people with infertility and RIF. Translocation was the main type of autosomal structural abnormalities (5 out of 6 observed abnormalities) (Figure 1a,b), accounting for 83.3% of these abnormalities. We also found one inversion (16.7% of all anomalies) (Figure 2) and one pstk ‐ (population polymorphism) (Figure 3).

The study was conducted on 200 healthy individuals who served as controls. Only one case of chromosome 9 inversion was found, which is a natural variation found in the population. This variation does not have any clinical symptoms. All the other participants had normal karyotypes. Therefore, the difference between the infertile and control groups was found to be statistically significant (*p* ≤ 0.05).

## 4 Discussion

This study investigated chromosomal abnormalities associated with RIF in western Iran for the first time. During our experiments, we examined 200 karyotypes in individuals who experienced RIF. Our results showed that six cases of chromosomal abnormalities were observed. Translocation was the main type of autosomal structural abnormality, accounting for 83.3% of these abnormalities (5 out of 6). We also found one inversion (16.7% of all anomalies) and one population polymorphism called pstk. Our findings suggest that these chromosomal anomalies in the fertilization process caused by IVF can result in a rise in abortions due to a genetic imbalance of the fetus caused by natural selection. Therefore, our study highlights the importance of pre‐implant genetic testing, which could prevent such cases.

Among the 200 individuals (100 couples) who had experienced RIF, a frequency of 3% karyotype anomalies was observed. The percentage reported in this study is different from that reported in some studies, which include 6.18% (Benard et al. 2019), 8.2% (Lee et al. 2019), or 1.3% in women and 1.5% in men (Neal et al. 2018).

According to literature data, the occurrence of balanced translocations in the general population is estimated to be within the range of 0.16%–0.2% (Gupta et al. 2019). However, our study discovered an approximately ten times higher rate of these translocations in the group of patients examined. Previous studies have suggested that there is a significantly elevated occurrence of balanced translocations in men experiencing infertility, ranging from five to ten times higher compared to the general population. In our study group, 4 males, which is around 4% of all males, were found to be carriers of balanced translocations. This finding is consistent with the results of Bojana Petrovic et al. In their study, two men out of 50 were carriers of balanced translocations (Petrovic et al. 2024).

Robertsonian translocations are a type of chromosomal abnormality that can cause severe spermatogenic dysfunction in infertile men (Chen and Zhou 2022). Studies have shown that these translocations are found in about 0.9%–3.4% of infertile men (Kuroda et al. 2020). However, in our study, none of the men were found to have Robertsonian translocations. In female patients, Robertsonian translocations appear to have minimal impact on gametogenesis. However, there is a higher risk of chromosome mosaicism during mitosis in blastocysts, which can lead to developmental abnormalities (Dang et al. 2023). In accordance with the study by Bojana Petrovic et al. (Petrovic et al. 2024), only one female was found to have a Robertsonian translocation.

The exact mechanism through which chromosomal abnormalities impact RIF remains unclear. Nevertheless, these translocations may interfere with crucial genes involved in embryo development, such as those regulating implantation and trophoblast function, gametogenesis, cell cycle regulation, or DNA repair processes (Ma et al. 2022).

Pericentric inversions are a type of structural chromosomal abnormality that occurs when a segment of chromosomal pieces is reversed in orientation relative to a reference karyotype. In a pericentric inversion, the segment that rotates contains the centromere (Puig et al. 2015). Individuals who have a pericentric inversion usually do not display any phenotypic characteristics unless there is genetic dysfunction or chromosomal damage present. Generally, inversion carriers have normal fertility potential. Pericentric inversions are mostly identified incidentally and are infrequently associated with infertility. However, if a crucial region on the chromosome is broken, adverse phenotypic changes can occur (Balasar et al. 2017).

Some individuals who carry inversions may experience reproductive issues due to abnormal meiotic events that result in chromosomally unbalanced gametes. In such cases, one can expect reproductive risks such as infertility, miscarriage, stillbirth, and the birth of malformed offspring (Balasar et al. 2017). The occurrence of pericentric inversions in men with azoospermia and oligozoospermia who are seeking IVF ranges from 0% to 0.3% (Mau‐Holzmann 2005). The risk of unbalanced chromosome rearrangement in blastocyst‐stage embryos from carriers of pericentric inversion of chromosome 1 is affected by inverted segment size (Jia and Xue 2023).

Our findings imply that these chromosomal anomalies in the fertilization process caused by IVF can lead to a rise in abortion, which is most likely related to pregnancy termination due to a genetic imbalance of the fetus caused by a natural selection process. These findings appear to be clinically meaningful, emphasizing the importance of pre‐implant genetic testing (Totonchi et al. 2021). Therefore, genetic counseling can help identify carriers of genetic transmission and provide families with information for making informed decisions regarding treatment continuation (Rodrigo et al. 2019).

Strengths of this study include the use of a well‐structured case–control design with a 1:1 matching ratio between case and control groups improving the statistical power of the comparisons. On the other hand, both partners in each couple underwent karyotyping, enabling a comprehensive cytogenetic assessment and identification of abnormalities in both sexes. Additionally, all chromosomal analyses were conducted using standardized G‐banding techniques, with examination of at least 20 metaphases per individual, ensuring diagnostic accuracy and methodological rigor. However, this study also had several limitations that should be considered when interpreting the findings. First, focusing on a specific region (Hamedan County) may limit the results' external validity to other populations. Second, selecting the control group from women presenting for reasons other than infertility may have introduced uncontrollable background differences from the case group. Third, the relatively low number of anomalies (only six cases in the RIF group) despite the census may limit further statistical analyses. Fourth, the voluntary presence of control men for karyotyping may lead to volunteer bias.

## 5 Conclusions

We found chromosomal abnormalities in 3% of study participants who had experienced RIF. Chromosomal abnormalities significantly contribute to RIF. Therefore, it is imperative to conduct cytogenetic screening for both partners before initiating any assisted reproductive technology procedures. By providing genetic counseling to couples, the patients who may benefit from preimplantation genetic diagnosis can be directed appropriately.

## Author Contributions

Atefeh Asgari and Amir Mohammad Salehi: Conceptualization. Safieh Ghahremani and Atefeh Asgari: methodology. Ebrahim Kamrani Saleh: software. Atefeh Asgari and Safieh Ghahremani: data curation. Amir Mohammad Salehi: writing – original draft preparation. Atefeh Asgari: writing – review and editing. All authors have read and agreed to the published version of the manuscript.

## Disclosure

Human and Animal Rights: No animals were used for studies that are the basis of this research. The reported experiments on couples are in accordance with the Helsinki Declaration of 1975, as revised in 2013 (https://ethics.iit.edu/ecodes/node/3931).

Standards of Reporting: STROBE guidelines were followed.

## Ethics Statement

The Ethics Committee of the Hamadan University of Medical Sciences approved the protocol of this study (IR.UMSHA.REC.1400.495).

## Consent

Consent was obtained before the study.

## Conflicts of Interest

The authors declare no conflicts of interest.

## Data Availability

The data that support the findings of this study are available from the corresponding author upon reasonable request.
